# Gestational timing and early neonatal outcomes in Palestine: a multicentre retrospective cohort study

**DOI:** 10.3389/fped.2026.1874317

**Published:** 2026-06-24

**Authors:** Motee Abuawwad, Mohammad Ibrahim Ghannam, Salahaldeen Deeb, Alaa Rashed Naji Said, Mohammed A. Taqatqah, Yousef Joulani, Bayan Abed Rabu, Ala Dwaib, Deema Alzeer, Salwa Sheikh Kasem, Hatem Mousa Khamash, Asmaa A. Rjoob

**Affiliations:** 1 Faculty of Medicine, Al-Quds University, Jerusalem, Palestine; 2 Department of Neonatology, Makassed Charitable Hospital, Jerusalem, Palestine; 3 Department of Neonatology, Al-Ahli Hospital, Hebron, Palestine; 4 Al-Ahli Hospital, Hebron, Palestine; 5 Medical Research Club, Al-Quds University, Jerusalem, Palestine

**Keywords:** early-term birth, elective caesarean section, late-preterm birth, neonatal morbidity, respiratory morbidity

## Abstract

**Introduction:**

Evidence on neonatal consequences of scheduling elective caesarean section before 39 completed weeks is limited in Palestine. We evaluated neonatal outcomes by delivery mode and gestational timing in two hospitals.

**Methods:**

This retrospective cohort study used records from Al-Makassed Charitable Hospital, Jerusalem, and Al-Ahli Hospital, Hebron, from January 2021 to December 2024. Liveborn singleton infants delivered at 34 + 0 weeks or later were included and grouped as elective caesarean section late preterm, elective caesarean section early term, non-assisted spontaneous vaginal delivery late preterm, non-assisted spontaneous vaginal delivery early term, or elective caesarean section at 39 + 0 weeks or later. The primary outcome was composite neonatal morbidity. Multivariable logistic regression adjusted for neonatal, maternal, and hospital covariates.

**Results:**

Among 8,364 infants, 3,347 were delivered by elective caesarean section; 2,569 (76.8%) occurred before 39 weeks. Composite morbidity was highest after late-preterm elective caesarean section (49.0%), followed by late-preterm vaginal birth (28.2%), early-term elective caesarean section (26.6%), elective caesarean section at 39 weeks or later (15.3%), and early-term vaginal birth (13.9%). Late-preterm elective caesarean section was independently associated with NICU admission, respiratory support, transient tachypnoea, respiratory distress syndrome requiring surfactant, hyperbilirubinaemia requiring treatment, and composite morbidity. Early-term elective caesarean section remained associated with composite morbidity.

**Conclusion:**

In this multicentre retrospective cohort, planned prelabour caesarean delivery before 39+0 weeks was associated with higher early neonatal morbidity, especially when performed in the late-preterm period. Because some late-preterm and early-term planned caesarean births may be medically indicated, these findings should be interpreted as support for careful documentation, senior review, and avoidance of non-medically indicated pre-39-week scheduling rather than as evidence that all earlier planned caesarean births should be deferred.

## Introduction

Caesarean section use has increased globally, with recent estimates showing that more than one fifth of births occur by caesarean section worldwide (1). Population-level caesarean rates alone do not distinguish between clinically necessary procedures and potentially avoidable early scheduling, and international guidance emphasizes that caesarean delivery can be lifesaving when medically indicated (2). The key neonatal concern is therefore not caesarean birth itself, but non-medically indicated delivery before 39 + 0 completed weeks when continuing pregnancy is clinically safe (3). When maternal, fetal, or placental indications exist, guidance also emphasizes that delivery before 39 + 0 weeks may be appropriate and should not be deferred solely to reach 39 weeks (4).

Gestational timing is central to this question because late-preterm birth is defined as 34 + 0 to 36 + 6 weeks and early-term birth as 37 + 0 to 38 + 6 weeks, while 39 + 0 weeks marks the beginning of the full-term period (5). For research and clinical documentation, gestational age should ideally reflect the best obstetric estimate rather than last menstrual period alone (6). Previous evidence shows a gestational-age gradient in adverse neonatal outcomes after planned caesarean birth, with higher morbidity at 37–38 weeks than at 39 weeks (7, 8). Prelabour caesarean birth may also increase respiratory morbidity because the absence of labour can delay fetal lung fluid clearance and cardiopulmonary transition (9).

Evidence from Palestine remains limited, although local estimates are needed because obstetric scheduling practice, case mix, and neonatal unit capacity differ across health systems. Regional data from Lebanon also suggest higher respiratory and other neonatal morbidity when term elective caesarean birth occurs before 39 weeks (10). This local context is important because the risk-benefit balance of planned early delivery may be shaped by referral pathways, documentation practices, and neonatal respiratory-support capacity. We therefore evaluated singleton infants born at or beyond 34 + 0 weeks in two Palestinian tertiary hospitals, comparing planned caesarean birth across late-preterm, early-term, and 39-week-or-later gestations and comparing planned caesarean birth with non-assisted spontaneous vaginal delivery at comparable earlier gestations. We hypothesised that planned prelabour caesarean birth before 39 + 0 weeks would be associated with higher early neonatal morbidity, while recognising that some earlier planned deliveries may have valid maternal or fetal indications.

## Methods

We conducted a multicentre retrospective cohort study using routinely collected electronic medical records from Al-Makassed Charitable Hospital, Jerusalem, and Al-Ahli Hospital, Hebron. The study period was 1 January 2021 to 31 December 2024. The sampling strategy was total enumeration: all delivery records during the study period were screened, and all records meeting the eligibility criteria were included; no random sampling was performed. Reporting followed the Strengthening the Reporting of Observational Studies in Epidemiology recommendations (11). Because this was an observational study using previously recorded routine clinical data, no clinical trial registration was required.

Eligible participants were liveborn singleton infants delivered at 34 + 0 weeks of gestation or later with sufficient obstetric and neonatal documentation to classify delivery mode, gestational age, and neonatal outcomes. Gestational age was extracted from the delivery record as the best obstetric estimate recorded by the treating obstetric team, based on available menstrual dating and antenatal ultrasound documentation. When available, early ultrasound-based dating was prioritised over last menstrual period alone, consistent with standard obstetric dating principles (6). We excluded births before 34 + 0 weeks, multiple gestations, non-elective or urgent caesarean births, assisted vaginal births, major congenital anomalies, intrauterine fetal death or stillbirth, and records with incomplete key variables. Gestational age categories were prespecified as late preterm (34 + 0–36 + 6 weeks), early term (37 + 0–38 + 6 weeks), and 39 + 0 weeks or later (5). Additional exclusion details are provided in online Supplementary Table S3 and Supplementary Figure S1.

The exposure was delivery strategy, defined by both delivery mode and gestational timing. Elective caesarean section (ECS) was defined operationally as a planned prelabour caesarean birth that was not documented as an emergency or urgent caesarean for acute maternal or fetal compromise. This operational category may include both non-medically indicated planned procedures and medically indicated scheduled procedures, such as repeat caesarean birth, placenta-related conditions, fetal growth concerns, hypertensive disease, diabetes-related indications, malpresentation, or maternal request/refusal of trial of labour after caesarean. We therefore use “ECS” to mean planned prelabour caesarean delivery, and we avoid interpreting all pre-39-week ECS as avoidable. Documented caesarean indications were extracted as non-mutually exclusive categories and are presented by gestational age group in online Supplementary Table S1.

Non-assisted spontaneous vaginal delivery (NSVD) referred to vaginal birth after spontaneous labour without operative assistance. Five analytic groups were defined *a priori*: ECS late preterm, ECS early term, NSVD late preterm, NSVD early term, and ECS at or beyond 39 + 0 weeks. ECS at or beyond 39 + 0 weeks was the reference group for analyses of planned caesarean timing. The primary analysis did not include NSVD at or beyond 39 + 0 weeks because detailed neonatal outcome abstraction was not performed for all term NSVD records during the screened period; therefore, the study should not be interpreted as comparing all groups with the lowest-risk physiological delivery reference.

Maternal variables included hospital site, age, gravidity, parity, gestational diabetes or abnormal oral glucose tolerance test, hypertensive disorder, hypothyroidism, thrombophilia, and documented caesarean indication. Neonatal baseline variables included sex, birth weight, Apgar scores at 1 and 5 min, and delivery-room resuscitation. When available, antenatal corticosteroid exposure was extracted, but this variable was not uniformly documented across both hospitals and was therefore not included in the primary pooled multivariable models.

Outcomes were harmonised across both hospitals using prespecified binary definitions before analysis. Structured fields were used when available; otherwise, diagnoses and discharge summaries were reviewed according to the same clinical definitions. The primary outcome was composite neonatal morbidity, defined as at least one recorded neonatal complication during the birth admission, NICU stay, or readmission window. Components included NICU admission, respiratory support, invasive respiratory support, transient tachypnoea of the newborn, respiratory distress syndrome requiring surfactant, hypothermia, hypoglycaemia, hyperbilirubinaemia requiring treatment, culture-positive sepsis, readmission within 28 days, neonatal death, and other major recorded neonatal morbidities such as hypotension, seizure requiring antiseizure medication, apnea requiring caffeine, intraventricular haemorrhage, necrotising enterocolitis, anaemia requiring transfusion, or polycythaemia requiring partial exchange transfusion. The composite was intended to quantify total early neonatal service burden and was not interpreted as a severity score. Component-specific outcomes and their frequencies are reported separately in Table 2 and online Supplementary Table S5.

Continuous variables were summarised as mean (standard deviation) or median (interquartile range), and categorical variables as number and percentage. Group comparisons used analysis of variance or Kruskal–Wallis tests for continuous variables and chi-square or Fisher exact tests for categorical variables. Crude risks were reported as n/N (%) for every outcome, and unadjusted relative risks with 95% confidence intervals were calculated for prespecified pairwise comparisons.

Because several outcomes, particularly NICU admission and composite neonatal morbidity, were common, adjusted odds ratios from logistic regression were not interpreted as risk ratios. The primary adjusted association measure was therefore the adjusted relative risk, estimated using modified Poisson regression with robust variance (12). Models adjusted for neonatal sex, maternal age, parity category, gestational diabetes or abnormal oral glucose tolerance test, hypertensive disorder, and hospital site. Birth weight was not included in the primary adjusted model because it may partly mediate the association between gestational age and neonatal morbidity; instead, sensitivity models were fitted with additional adjustment for birth weight, and birthweight-for-gestational-age category was used when available (13). Logistic regression models were retained as sensitivity analyses and are reported as adjusted odds ratios in the Supplementary Material.

Missingness was assessed for each exposure, covariate, and outcome and is reported in online Supplementary Table S4. Complete-case analysis was used for the primary adjusted models because exposure and outcome classification depended on contemporaneous clinical documentation. The number of observations included in each adjusted model is reported in the corresponding table. If a prespecified model failed to converge or had sparse cells, crude risks and unadjusted relative risks were reported and unstable adjusted models were not fitted.

## Results

The analytic cohort included 8,364 eligible singleton births: 2,560 from Al-Makassed and 5,804 from Al-Ahli. The five prespecified groups were ECS late preterm (*n* = 261), ECS early term (*n* = 2,308), NSVD late preterm (*n* = 581), NSVD early term (*n* = 4,436), and ECS at or beyond 39 weeks (*n* = 778). Among 3,347 elective caesarean births, 2,569 (76.8%) occurred before 39 completed weeks.

During the study period, all delivery records from both hospitals were screened using the prespecified eligibility criteria. The main reasons for exclusion were NSVD at or beyond 39 + 0 weeks, non-elective or urgent delivery, multiple gestation, gestational age below 34 + 0 weeks, congenital anomaly or malformation, fetal/neonatal death before live-birth eligibility, assisted vaginal delivery, and incomplete or inconsistent key documentation (online Supplementary Table S3; Supplementary Figure S1).

Baseline characteristics differed across groups (Table 1). Mothers in ECS groups were older and had higher gravidity and parity than mothers in NSVD groups. Gestational diabetes, hypertensive disorders, hypothyroidism, and thrombophilia were more frequent in the pre-39-week ECS groups. Birth weight increased with gestational age, from a mean of 2,702.6 g in ECS late-preterm infants to 3,470.8 g in the ECS at or beyond 39 weeks group. One-minute Apgar score below 7 and delivery-room resuscitation were most common after late-preterm ECS, suggesting greater transitional vulnerability in this group at birth. Sex distribution was broadly similar, although male infants were slightly more frequent in the late-preterm groups.

**Table 1:** Maternal and neonatal baseline characteristics across prespecified delivery-strategy groups.

Variable	ECS 34 + 0–36 + 6 (*n* = 261)	NSVD 34 + 0–36 + 6 (*n* = 581)	ECS 37 + 0–38 + 6 (*n* = 2,308)	NSVD 37 + 0–38 + 6 (*n* = 4,436)	ECS >=39 + 0 (*n* = 778)	*p* value
Hospital site: Al-Makassed	145/261 (55.6%)	158/581 (27.2%)	952/2,308 (41.2%)	1,033/4,436 (23.3%)	272/778 (35.0%)	<0.001
Hospital site: Al-Ahli	116/261 (44.4%)	423/581 (72.8%)	1,356/2,308 (58.8%)	3,403/4,436 (76.7%)	506/778 (65.0%)	
Male sex	152/261 (58.2%)	328/581 (56.5%)	1,210/2,308 (52.4%)	2,387/4,436 (53.8%)	390/778 (50.1%)	0.057
Maternal age, years	31.2 +/- 5.9	25.8 +/- 5.7	30.4 +/- 5.5	26.5 +/- 5.4	28.2 +/- 5.8	<0.001
Gravidity	4 [3–6]	3 [2–4]	4 [3–5]	3 [2–4]	3 [2–5]	<0.001
Primigravida	16/261 (6.1%)	133/581 (22.9%)	165/2,308 (7.1%)	937/4,436 (21.1%)	126/778 (16.2%)	<0.001
Multigravida	244/261 (93.5%)	444/581 (76.4%)	2,125/2,308 (92.1%)	3,475/4,436 (78.3%)	648/778 (83.3%)	<0.001
Parity	4 [2–5]	2 [1–4]	3 [2–4]	2 [1–4]	2 [1–4]	<0.001
Primipara	30/261 (11.5%)	162/581 (27.9%)	252/2,308 (10.9%)	1,136/4,436 (25.6%)	196/778 (25.2%)	<0.001
Multipara	217/261 (83.1%)	384/581 (66.1%)	1,964/2,308 (85.1%)	2,966/4,436 (66.9%)	546/778 (70.2%)	<0.001
Diabetes or abnormal OGTT	26/261 (10.0%)	16/581 (2.8%)	220/2,308 (9.5%)	129/4,436 (2.9%)	45/778 (5.8%)	<0.001
Hypertensive disorder	20/261 (7.7%)	13/581 (2.2%)	96/2,308 (4.2%)	91/4,436 (2.1%)	14/778 (1.8%)	<0.001
Hypothyroidism	21/261 (8.0%)	22/581 (3.8%)	126/2,308 (5.5%)	121/4,436 (2.7%)	32/778 (4.1%)	<0.001
Thrombophilia	18/261 (6.9%)	21/581 (3.6%)	172/2,308 (7.5%)	246/4,436 (5.5%)	16/778 (2.1%)	<0.001
Birth weight, g	2,702.6 +/- 507.1	2,739.9 +/- 366.1	3,108.6 +/- 419.2	3,058.0 +/- 378.8	3,470.8 +/- 490.3	<0.001
Apgar score at 1 min	8 [7–8]	8 [8–8]	8 [8–9]	8 [8–8]	8 [8–9]	<0.001
Apgar score at 1 min <7	50/261 (19.2%)	15/581 (2.6%)	217/2,308 (9.4%)	45/4,436 (1.0%)	36/778 (4.6%)	<0.001
Apgar score at 5 min	9 [9–9]	9 [9–9]	9 [9–9]	9 [9–9]	9 [9–9]	<0.001
Apgar score at 5 min <7	8/261 (3.1%)	0/581 (0.0%)	23/2,308 (1.0%)	5/4,436 (0.1%)	3/778 (0.4%)	<0.001
Delivery-room resuscitation	73/261 (28.0%)	48/581 (8.3%)	172/2,308 (7.5%)	40/4,436 (0.9%)	53/778 (6.8%)	<0.001

Data are n/N (%) for categorical variables, mean +/- standard deviation for normally distributed continuous variables, or median [interquartile range] for skewed variables. Percentages use the full group denominator shown in the column heading unless otherwise indicated. *p* values are from chi-square or Fisher exact tests for categorical variables, one-way analysis of variance for normally distributed variables, and Kruskal–Wallis tests for skewed variables. ECS, elective caesarean section; NSVD, non-assisted spontaneous vaginal delivery; OGTT, oral glucose tolerance test.

Documented ECS indications are presented by gestational age category in online Supplementary Table S1. Indication categories were not mutually exclusive. Previous caesarean section was the most common indication overall, followed by maternal request or refusal of trial of labour after caesarean, other/unspecified indications, suspected macrosomia or large-for-gestational-age fetus, fetal growth restriction/small-for-gestational-age concerns, diabetes or abnormal oral glucose tolerance test, hypertensive disorders, PPROM or oligohydramnios, short interpregnancy interval, and planned tubal ligation.

Maternal glycaemic status differed across delivery-strategy groups. Gestational diabetes or abnormal oral glucose tolerance test was recorded in 26/261 (10.0%) of ECS late-preterm births, 220/2,308 (9.5%) of ECS early-term births, 45/778 (5.8%) of ECS at or beyond 39 + 0 weeks, 16/581 (2.8%) of NSVD late-preterm births, and 129/4,436 (2.9%) of NSVD early-term births (Table 1). Because maternal glycaemic status may influence neonatal hypoglycaemia, respiratory morbidity, and NICU admission, it was retained in all adjusted models.

**Figure 1:**
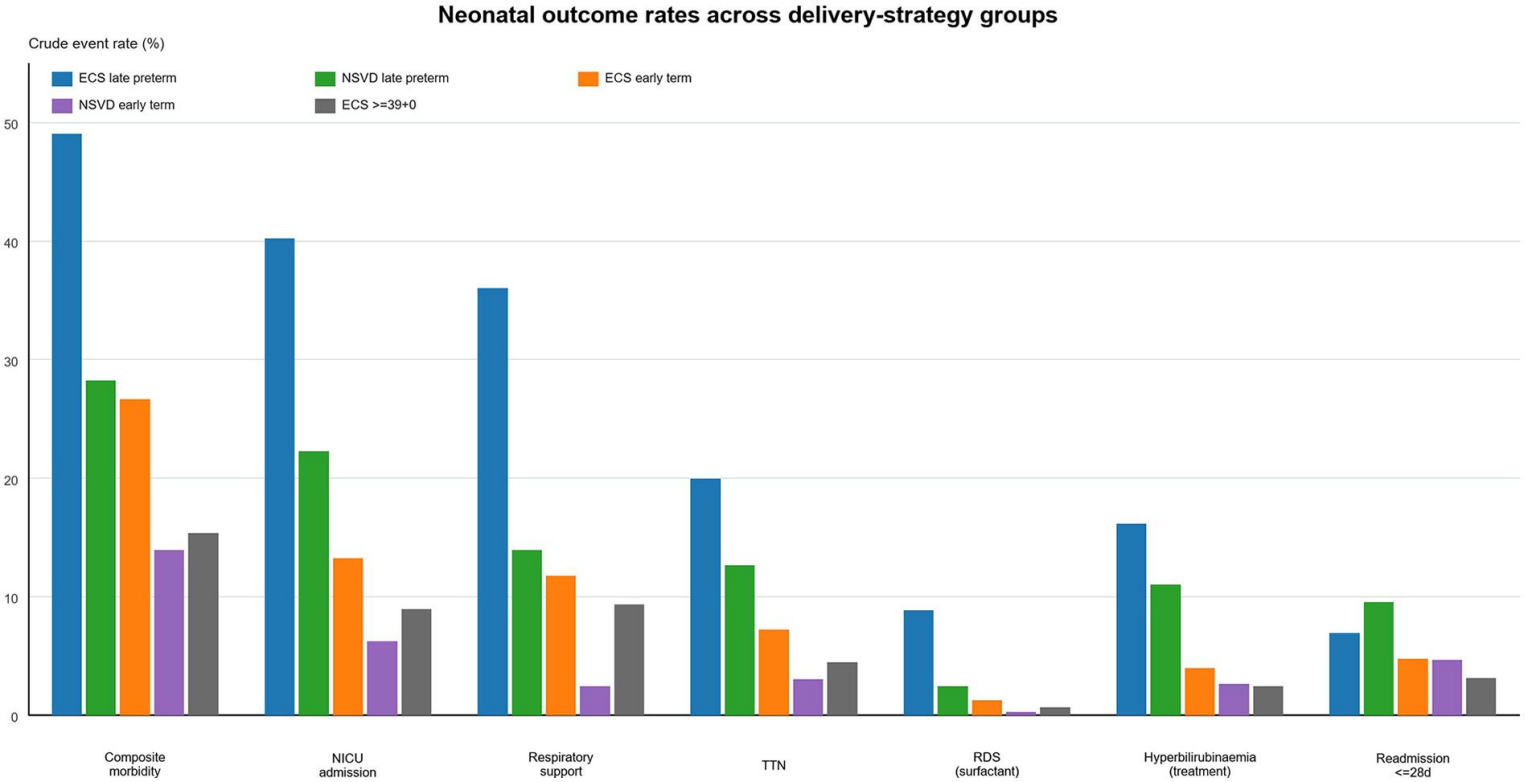
Neonatal outcome rates across delivery-strategy groups. Bars show crude event rates (%) for ECS late preterm, NSVD late preterm, ECS early term, NSVD early term, and ECS at or beyond 39 + 0 weeks. This figure aligns with the prespecified analytic groups and should be interpreted together with crude event counts in Table 2.

Composite neonatal morbidity was highest after late-preterm ECS, affecting 128 of 261 infants (49.0%), followed by late-preterm NSVD (28.2%), early-term ECS (26.6%), ECS at or beyond 39 weeks (15.3%), and early-term NSVD (13.9%) (Table 2; Figure 1). NICU admission showed the same gradient, occurring in 40.2% of late-preterm ECS infants compared with 13.2% after early-term ECS, 22.2% after late-preterm NSVD, 6.2% after early-term NSVD, and 8.9% after ECS at or beyond 39 weeks.

**Table 2:** Early neonatal outcomes across prespecified delivery-strategy groups.

Variable	ECS 34 + 0–36 + 6 (*n* = 261)	NSVD 34 + 0–36 + 6 *n* = 581)	ECS 37 + 0–38 + 6 (*n* = 2,308)	NSVD 37 + 0–38 + 6(*n* = 4,436)	ECS >=39 + 0 (*n* = 778)	*p* value
Composite neonatal morbidity	128/261 (49.0%)	164/581 (28.2%)	613/2,308 (26.6%)	616/4,436 (13.9%)	119/778 (15.3%)	<0.001
NICU admission	105/261 (40.2%)	129/581 (22.2%)	304/2,308 (13.2%)	273/4,436 (6.2%)	69/778 (8.9%)	<0.001
NICU length of stay, days^a^	4 [2–8]	3.0 [2.0–5.2]	3 [2–4]	2 [2–4]	3.0 [2.0–3.2]	<0.001
Delivery-room resuscitation	73/261 (28.0%)	48/581 (8.3%)	172/2,308 (7.5%)	40/4,436 (0.9%)	53/778 (6.8%)	<0.001
Any respiratory support	94/261 (36.0%)	81/581 (13.9%)	270/2,308 (11.7%)	108/4,436 (2.4%)	72/778 (9.3%)	<0.001
Invasive respiratory support	11/261 (4.2%)	4/581 (0.7%)	14/2,308 (0.6%)	8/4,436 (0.2%)	8/778 (1.0%)	<0.001
Transient tachypnoea of the newborn	52/261 (19.9%)	73/581 (12.6%)	166/2,308 (7.2%)	132/4,436 (3.0%)	34/778 (4.4%)	<0.001
RDS requiring surfactant	23/261 (8.8%)	14/581 (2.4%)	27/2,308 (1.2%)	10/4,436 (0.2%)	5/778 (0.6%)	<0.001
Hypothermia	62/261 (23.8%)	76/581 (13.1%)	379/2,308 (16.4%)	404/4,436 (9.1%)	73/778 (9.4%)	<0.001
Hypoglycaemia	6/261 (2.3%)	10/581 (1.7%)	39/2,308 (1.7%)	34/4,436 (0.8%)	4/778 (0.5%)	<0.001
Hypotension	1/261 (0.4%)	1/581 (0.2%)	1/2,308 (0.0%)	1/4,436 (0.0%)	1/778 (0.1%)	0.110
Anaemia requiring transfusion	12/261 (4.6%)	4/581 (0.7%)	3/2,308 (0.1%)	2/4,436 (0.0%)	1/778 (0.1%)	<0.001
Polycythaemia requiring exchange transfusion	10/261 (3.8%)	1/581 (0.2%)	8/2,308 (0.3%)	3/4,436 (0.1%)	3/778 (0.4%)	<0.001
Hyperbilirubinaemia requiring treatment	42/261 (16.1%)	64/581 (11.0%)	90/2,308 (3.9%)	115/4,436 (2.6%)	19/778 (2.4%)	<0.001
Culture-positive sepsis	3/261 (1.1%)	4/581 (0.7%)	2/2,308 (0.1%)	3/4,436 (0.1%)	0/778 (0.0%)	<0.001
Intraventricular haemorrhage	2/261 (0.8%)	4/581 (0.7%)	1/2,308 (0.0%)	1/4,436 (0.0%)	0/778 (0.0%)	<0.001
Apnoea requiring caffeine	3/261 (1.1%)	2/581 (0.3%)	6/2,308 (0.3%)	4/4,436 (0.1%)	1/778 (0.1%)	0.003
Seizure requiring antiseizure medication	0/261 (0.0%)	0/581 (0.0%)	0/2,308 (0.0%)	1/4,436 (0.0%)	0/778 (0.0%)	0.927
Necrotising enterocolitis	2/261 (0.8%)	3/581 (0.5%)	2/2,308 (0.1%)	0/4,436 (0.0%)	0/778 (0.0%)	<0.001
Readmission within 28 days	18/261 (6.9%)	55/581 (9.5%)	108/2,308 (4.7%)	205/4,436 (4.6%)	24/778 (3.1%)	<0.001
Neonatal death	1/261 (0.4%)	1/581 (0.2%)	1/2,308 (0.0%)	2/4,436 (0.0%)	2/778 (0.3%)	0.117

a NICU length of stay is summarised among infants admitted to NICU and reported as median [interquartile range]. Composite neonatal morbidity denotes at least one recorded neonatal morbidity or service-use outcome during the birth admission, NICU stay, or 28-day readmission window. ECS, elective caesarean section; NICU, neonatal intensive care unit; NSVD, non-assisted spontaneous vaginal delivery; RDS, respiratory distress syndrome; TTN, transient tachypnoea of the newborn.

The components of the composite endpoint are shown separately in online Supplementary Table S5. The largest contributors to composite morbidity were NICU admission, respiratory morbidity, hypothermia, hyperbilirubinaemia requiring treatment, and readmission within 28 days. Findings were directionally similar when the composite was separated into a respiratory composite and a non-respiratory composite, although precision was lower for rarer outcomes.

Respiratory morbidity was also concentrated in earlier ECS groups. Any respiratory support was required in 36.0% of late-preterm ECS infants and 11.7% of early-term ECS infants, compared with 9.3% after ECS at or beyond 39 weeks. TTN occurred in 19.9%, 7.2%, and 4.4% of these groups, respectively, and RDS requiring surfactant occurred in 8.8%, 1.2%, and 0.6%, respectively. Hypothermia and hyperbilirubinaemia requiring treatment were most frequent in the late-preterm ECS group. Neonatal death, seizure requiring antiseizure medication, and hypotension were rare and did not differ significantly across groups.

In delivery-strategy comparisons, ECS before 39 weeks carried higher unadjusted risk than ECS at or beyond 39 weeks for NICU admission (RR 1.80, 95% CI 1.41–2.29), any respiratory support (RR 1.53, 95% CI 1.20–1.95), TTN (RR 1.94, 95% CI 1.37–2.76), RDS requiring surfactant (RR 3.03, 95% CI 1.21–7.57), and composite morbidity (RR 1.89, 95% CI 1.58–2.25) (Table 3). Compared with vaginal birth before 39 weeks, ECS before 39 weeks was associated with higher risk of any respiratory support (RR 3.76, 95% CI 3.18–4.45), TTN (RR 2.08, 95% CI 1.73–2.50), RDS requiring surfactant (RR 4.07, 95% CI 2.51–6.60), and composite morbidity (RR 1.86, 95% CI 1.70–2.03).

**Table 3:** Unadjusted relative risks for key neonatal outcomes in collapsed delivery-strategy comparisons.

Outcome	ECS <39 + 0 (*n* = 2,569)	ECS >=39 + 0 (*n* = 778)	RR (95% CI) ECS <39 vs. ECS >=39	*p* value	NSVD <39 + 0 (*n* = 5,017)	RR (95% CI) ECS <39 vs. NSVD <39	*p* value
NICU admission	409/2,569 (15.9%)	69/778 (8.9%)	1.80 (1.41–2.29)	<0.001	402/5,017 (8.0%)	1.99 (1.75–2.26)	<0.001
Any respiratory support	364/2,569 (14.2%)	72/778 (9.3%)	1.53 (1.20–1.95)	<0.001	189/5,017 (3.8%)	3.76 (3.18–4.45)	<0.001
Invasive respiratory support	25/2,569 (1.0%)	8/778 (1.0%)	0.95 (0.43–2.09)	0.892	12/5,017 (0.2%)	4.07 (2.05–8.08)	<0.001
Transient tachypnoea of the newborn	218/2,569 (8.5%)	34/778 (4.4%)	1.94 (1.37–2.76)	<0.001	205/5,017 (4.1%)	2.08 (1.73–2.50)	<0.001
RDS requiring surfactant	50/2,569 (1.9%)	5/778 (0.6%)	3.03 (1.21–7.57)	0.012	24/5,017 (0.5%)	4.07 (2.51–6.60)	<0.001
Composite neonatal morbidity	741/2,569 (28.8%)	119/778 (15.3%)	1.89 (1.58–2.25)	<0.001	780/5,017 (15.5%)	1.86 (1.70–2.03)	<0.001
Hypothermia	441/2,569 (17.2%)	73/778 (9.4%)	1.83 (1.45–2.31)	<0.001	480/5,017 (9.6%)	1.79 (1.59–2.02)	<0.001
Hypoglycaemia	45/2,569 (1.8%)	4/778 (0.5%)	3.41 (1.23–9.44)	0.012	44/5,017 (0.9%)	2.00 (1.32–3.02)	<0.001
Hyperbilirubinaemia requiring treatment	132/2,569 (5.1%)	19/778 (2.4%)	2.10 (1.31–3.38)	0.002	179/5,017 (3.6%)	1.44 (1.16–1.79)	0.001
Readmission within 28 days	126/2,569 (4.9%)	24/778 (3.1%)	1.59 (1.04–2.44)	0.032	260/5,017 (5.2%)	0.95 (0.77–1.16)	0.602

RRs are unadjusted and compare crude risks between the stated exposure and reference groups. ECS <39 + 0 includes ECS at 34 + 0–38 + 6 weeks. NSVD <39 + 0 includes NSVD at 34 + 0–38 + 6 weeks. ECS, elective caesarean section; NSVD, non-assisted spontaneous vaginal delivery; RR, relative risk; CI, confidence interval; RDS, respiratory distress syndrome; TTN, transient tachypnoea of the newborn.

**Figure 2:**
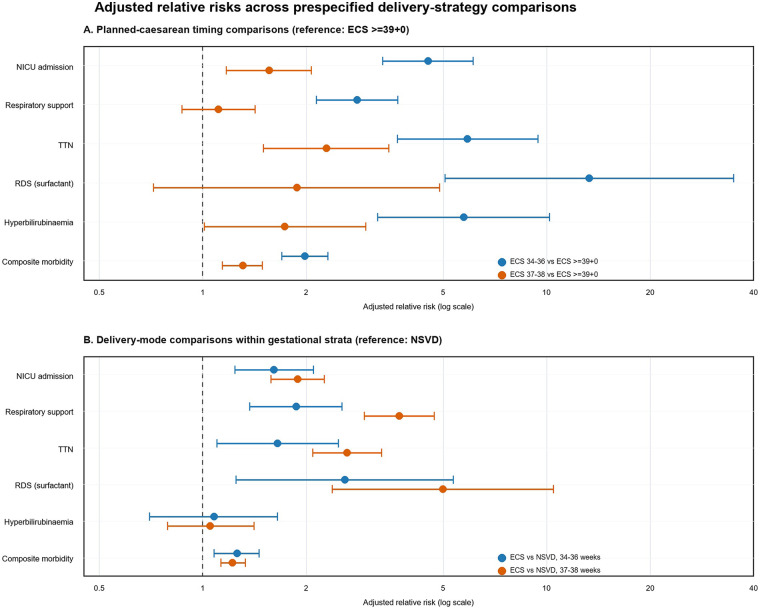
Adjusted relative risks for key neonatal outcomes across prespecified delivery-strategy comparisons. **(A)** Planned-caesarean timing comparisons, using ECS at or beyond 39 + 0 weeks as the reference group. **(B)** Delivery-mode comparisons within gestational-age strata, using NSVD in the same gestational-age stratum as the reference group. Points show adjusted relative risks with 95% confidence intervals from modified Poisson models with robust variance. The vertical dashed line indicates no association.

In modified Poisson models, late-preterm ECS remained associated with higher adjusted risk of NICU admission (adjusted relative risk 4.52, 95% CI 3.34–6.12), respiratory support (adjusted relative risk 2.81, 95% CI 2.14–3.69), TTN (adjusted relative risk 5.89, 95% CI 3.68–9.43), RDS requiring surfactant (adjusted relative risk 13.30, 95% CI 5.06–34.95), hyperbilirubinaemia requiring treatment (adjusted relative risk 5.74, 95% CI 3.23–10.20), and composite morbidity (adjusted relative risk 1.98, 95% CI 1.70–2.31) compared with ECS at or beyond 39 + 0 weeks (Table 4, Figure 2). Early-term ECS remained associated with NICU admission (adjusted relative risk 1.56, 95% CI 1.17–2.07), TTN (adjusted relative risk 2.29, 95% CI 1.50–3.48), hyperbilirubinaemia requiring treatment (adjusted relative risk 1.73, 95% CI 1.01–2.98), and composite morbidity (adjusted relative risk 1.31, 95% CI 1.14–1.49), while associations with respiratory support and RDS requiring surfactant were smaller or less precise after covariate adjustment. All primary adjusted models included 7,959 complete cases. Sensitivity analyses adding birth weight attenuated the estimates, particularly for composite morbidity and respiratory support, but late-preterm ECS remained associated with higher risk across the main outcomes (online Supplementary Table S6). Because antenatal corticosteroid data were not uniformly available across hospitals, this variable was not included in the primary pooled models. A pooled corticosteroid subgroup analysis was not performed because the exposure was unavailable or inconsistently documented for a substantial proportion of records, creating a high risk of selection and documentation bias.

**Table 4:** Primary adjusted relative risks from modified poisson regression for planned caesarean timing.

Outcome	ECS 34 + 0–36 + 6 vs. ECS >=39 + 0 aRR (95% CI)	*p* value	ECS 37 + 0–38 + 6 vs. ECS >=39 + 0 aRR (95% CI)	*p* value	Model N
NICU admission	4.52 (3.34–6.12)	<0.001	1.56 (1.17–2.07)	0.002	7,959
Any respiratory support	2.81 (2.14–3.69)	<0.001	1.11 (0.87–1.42)	0.404	7,959
Transient tachypnoea of the newborn	5.89 (3.68–9.43)	<0.001	2.29 (1.50–3.48)	<0.001	7,959
RDS requiring surfactant	13.30 (5.06–34.95)	<0.001	1.88 (0.72–4.89)	0.196	7,959
Hyperbilirubinaemia requiring treatment	5.74 (3.23–10.20)	<0.001	1.73 (1.01–2.98)	0.047	7,959
Composite neonatal morbidity	1.98 (1.70–2.31)	<0.001	1.31 (1.14–1.49)	<0.001	7,959

Adjusted relative risks were estimated using modified Poisson regression with robust variance. The reference group was ECS at or beyond 39 + 0 weeks. Primary models adjusted for neonatal sex, maternal age, parity category, diabetes or abnormal OGTT, hypertensive disorder, and hospital site. Birth weight was not included in the primary model because it may partly mediate the association between gestational timing and neonatal morbidity. aRR, adjusted relative risk; CI, confidence interval; ECS, elective caesarean section; OGTT, oral glucose tolerance test; RDS, respiratory distress syndrome; TTN, transient tachypnoea of the newborn.

## Discussion

In this multicentre Palestinian cohort, planned prelabour caesarean delivery before 39 + 0 completed weeks was associated with higher early neonatal morbidity than planned caesarean delivery at or beyond 39 + 0 weeks. The association was strongest in the late-preterm period, where almost half of infants had at least one recorded morbidity and 40% were admitted to NICU. These findings are consistent with international evidence showing a gestational-age gradient in neonatal morbidity after planned caesarean delivery (7, 8). They also add local evidence from Palestine, where neonatal unit capacity and obstetric scheduling pathways may differ from higher-income settings.

The results also show that route of birth contributed to risk beyond gestational age alone. Within the early-term stratum, ECS was associated with higher adjusted odds of NICU admission, respiratory support, TTN, RDS requiring surfactant, and composite morbidity than NSVD. This pattern is biologically plausible because labour promotes catecholamine release, epithelial sodium transport, and lung fluid absorption, whereas prelabour caesarean delivery may delay pulmonary transition (7, 8). The magnitude of the respiratory association is clinically important for hospitals with constrained NICU capacity because even transient respiratory illness can require monitoring, oxygen, non-invasive ventilation, and separation of mother and infant.

However, the interpretation must account for confounding by indication. Some planned caesarean births before 39 + 0 weeks are scheduled because continuing pregnancy may increase maternal or fetal risk. Therefore, our findings should not be read as evidence that all planned pre-39-week caesarean births should be postponed. The clinically relevant distinction is between medically indicated earlier birth and non-medically indicated early scheduling (3, 4).

Our early-term ECS findings were more nuanced than the late-preterm results. Crude analyses showed higher rates of NICU admission, TTN, hypothermia, and composite morbidity after early-term ECS compared with ECS at or beyond 39 + 0 weeks, but adjusted estimates were smaller. This suggests that part of the early-term association may be explained by differences in maternal risk profile, hospital site, and infant size. Birth weight requires cautious interpretation because it may be a marker of fetal growth restriction, a confounder of indicated delivery, or a mediator between shorter gestation and neonatal morbidity (13). For this reason, we present models with and without birth-weight adjustment rather than treating birth weight as a simple confounder.

More than three quarters of ECS births in this cohort occurred before 39 weeks. This high proportion identifies a practical target for quality improvement. Previous caesarean birth was the most common documented indication, indicating that repeat caesarean scheduling is a major driver of exposure. Reducing avoidable early ECS will require clear local scheduling criteria, explicit documentation of medical indications, and counselling that explains early-term neonatal risk without dismissing valid maternal or fetal indications for earlier delivery (3, 4). Similar regional evidence suggests that postponing elective caesarean birth until 39 weeks may prevent a meaningful proportion of respiratory morbidity (12). Audit tools could include a mandatory booking field for indication, a hard-stop review for pre-39-week elective scheduling, and monthly feedback on NICU admissions following planned caesarean birth. Such measures should be paired with rapid access pathways for women who develop labour, bleeding, hypertension, ruptured membranes, or fetal concerns before the scheduled date.

Implementation should not be interpreted as a rigid prohibition of earlier delivery. Some pregnancies require late-preterm or early-term birth to protect the mother or fetus, and delaying such deliveries could be harmful. The practical distinction is between indicated and non-indicated early scheduling. A local policy should therefore combine neonatal risk counselling with senior obstetric review, documentation of the indication, and shared decision-making that records why the selected gestational age is safer than waiting until 39 weeks.

The study has several strengths. It included a large contemporary cohort from two tertiary hospitals, used prespecified gestational categories, compared ECS both with later ECS and with NSVD at similar gestations, and adjusted for important maternal, neonatal, and hospital-level covariates. Harmonisation of outcome definitions allowed pooled analysis across two routine data systems. The inclusion of both relative-risk estimates and adjusted models improves interpretability: crude estimates describe the service burden, whereas adjusted estimates address whether associations persist after accounting for measured differences in infant size, maternal risk, and hospital site.

The study has limitations. Its retrospective design leaves residual confounding by indication possible, especially because detailed severity of hypertensive disease, placenta previa/accreta spectrum, fetal growth restriction severity, prelabour rupture of membranes, maternal body mass index, smoking, antenatal care adequacy, and some fetal indications were not uniformly available. Antenatal corticosteroid exposure was also not consistently documented across both hospitals, preventing a reliable pooled subgroup analysis. This is important because corticosteroids can reduce respiratory morbidity in selected late-preterm births, although evidence around routine use before planned term caesarean birth is more nuanced (14, 15). Missing data were handled by complete-case analysis, and missingness is now reported explicitly; nevertheless, documentation gaps may have introduced selection bias. The cohort did not include detailed outcome abstraction for NSVD at or beyond 39 + 0 weeks, so ECS at or beyond 39 + 0 weeks was the reference for planned caesarean timing rather than a lowest-risk physiological reference group. Finally, rare outcomes such as neonatal death, necrotising enterocolitis, culture-positive sepsis, seizure, and invasive ventilation had limited precision.

These limitations mean that the findings should be interpreted as associations rather than proof of a causal effect of timing alone. The results support local audit of planned pre-39-week caesarean births, explicit documentation of medical indications, and multidisciplinary review when earlier delivery is scheduled without a clear maternal or fetal indication. Such audit may help reduce avoidable neonatal morbidity and NICU use, but decisions for individual pregnancies should remain based on the balance of maternal, fetal, and neonatal risks.

## Conclusion

Planned prelabour caesarean delivery before 39 + 0 completed weeks, particularly during the late-preterm period, was associated with higher early neonatal morbidity, respiratory support, and NICU admission in two Palestinian tertiary hospitals. Both gestational timing and delivery mode contributed to risk, but residual confounding by indication remains possible. These findings support careful documentation and review of pre-39-week planned caesarean scheduling and avoidance of earlier delivery when there is no clear maternal or fetal indication. They should not be interpreted as recommending delay when earlier birth is medically indicated.

## Data Availability

The raw data supporting the conclusions of this article will be made available by the authors, without undue reservation.
