# Supplementary material for: Gestational timing and early neonatal outcomes in Palestine: a multicentre retrospective cohort study
Source: Front Pediatr. 2026 Jun 24;14:1874317. doi: 10.3389/fped.2026.1874317 (PMC13341800; doi:10.3389/fped.2026.1874317)
Supplement: Supplementary file 1 [file Supplementaryfile1.docx]

**Online Supplementary Material**

**Gestational Timing of Elective Caesarean Delivery and Early Neonatal Outcomes in Palestine: A Multicentre Retrospective Cohort Study**

**Table S1. Documented indications for elective caesarean section by gestational-age group**

| **Indication** | **ECS 34+0–36+6** | **ECS 37+0–38+6** | **ECS ≥39+0** | **Overall ECS** | **p value** |
| --- | --- | --- | --- | --- | --- |
| Previous CS | 176/261 (67.4%) | 1842/2308 (79.8%) | 452/778 (58.1%) | 2470/3347 (73.8%) | <0.001 |
| Maternal request / TOLAC refusal | 13/261 (5.0%) | 280/2308 (12.1%) | 184/778 (23.7%) | 477/3347 (14.3%) | <0.001 |
| Suspected macrosomia/LGA | 2/261 (0.8%) | 31/2308 (1.3%) | 107/778 (13.8%) | 140/3347 (4.2%) | <0.001 |
| IUGR/SGA/FGR | 24/261 (9.2%) | 55/2308 (2.4%) | 3/778 (0.4%) | 82/3347 (2.4%) | <0.001 |
| Diabetes / abnormal OGTT | 11/261 (4.2%) | 121/2308 (5.2%) | 28/778 (3.6%) | 160/3347 (4.8%) | 0.161 |
| Hypertensive disorder | 11/261 (4.2%) | 40/2308 (1.7%) | 2/778 (0.3%) | 53/3347 (1.6%) | <0.001 |
| Placenta previa/accreta spectrum | 37/261 (14.2%) | 32/2308 (1.4%) | 1/778 (0.1%) | 70/3347 (2.1%) | <0.001 |
| PPROM / oligohydramnios | 16/261 (6.1%) | 24/2308 (1.0%) | 20/778 (2.6%) | 60/3347 (1.8%) | <0.001 |
| Short interval | 0/261 (0.0%) | 25/2308 (1.1%) | 12/778 (1.5%) | 37/3347 (1.1%) | 0.117 |
| Tubal ligation | 2/261 (0.8%) | 10/2308 (0.4%) | 1/778 (0.1%) | 13/3347 (0.4%) | 0.295 |
| Other/unspecified | 33/261 (12.6%) | 238/2308 (10.3%) | 134/778 (17.2%) | 405/3347 (12.1%) | <0.001 |

Note. Indication categories are not mutually exclusive because a single planned caesarean birth could have more than one documented indication. Percentages are calculated using all ECS births within each gestational-age group as the denominator. P values compare indication frequency across the three ECS gestational-age groups.

**Table S2. Prespecified pairwise relative-risk comparisons**

| **Comparison** | **Outcome** | **Exposure** | **Reference** | **RR (95% CI)** | **p** |
| --- | --- | --- | --- | --- | --- |
| ECS early term vs ECS ≥39 | NICU admission | 304/2308 (13.2%) | 69/778 (8.9%) | 1.49 (1.16-1.90) | 0.001 |
| ECS early term vs ECS ≥39 | Any respiratory support | 270/2308 (11.7%) | 72/778 (9.3%) | 1.26 (0.99-1.62) | 0.060 |
| ECS early term vs ECS ≥39 | Invasive respiratory support | 14/2308 (0.6%) | 8/778 (1.0%) | 0.59 (0.25-1.40) | 0.227 |
| ECS early term vs ECS ≥39 | Transient tachypnea of the newborn | 166/2308 (7.2%) | 34/778 (4.4%) | 1.65 (1.15-2.36) | 0.006 |
| ECS early term vs ECS ≥39 | RDS requiring surfactant | 27/2308 (1.2%) | 5/778 (0.6%) | 1.82 (0.70-4.71) | 0.209 |
| ECS early term vs ECS ≥39 | Any morbidity | 613/2308 (26.6%) | 119/778 (15.3%) | 1.74 (1.45-2.08) | <0.001 |
| ECS early term vs ECS ≥39 | Hypothermia | 379/2308 (16.4%) | 73/778 (9.4%) | 1.75 (1.38-2.22) | <0.001 |
| ECS early term vs ECS ≥39 | Hypoglycemia | 39/2308 (1.7%) | 4/778 (0.5%) | 3.29 (1.18-9.17) | 0.016 |
| ECS early term vs ECS ≥39 | Hyperbilirubinemia requiring treatment | 90/2308 (3.9%) | 19/778 (2.4%) | 1.60 (0.98-2.60) | 0.057 |
| ECS early term vs ECS ≥39 | Readmission within 28 days | 108/2308 (4.7%) | 24/778 (3.1%) | 1.52 (0.98-2.34) | 0.057 |
| ECS late preterm vs ECS ≥39 | NICU admission | 105/261 (40.2%) | 69/778 (8.9%) | 4.54 (3.46-5.94) | <0.001 |
| ECS late preterm vs ECS ≥39 | Any respiratory support | 94/261 (36.0%) | 72/778 (9.3%) | 3.89 (2.96-5.11) | <0.001 |
| ECS late preterm vs ECS ≥39 | Invasive respiratory support | 11/261 (4.2%) | 8/778 (1.0%) | 4.10 (1.67-10.08) | 0.002 |
| ECS late preterm vs ECS ≥39 | Transient tachypnea of the newborn | 52/261 (19.9%) | 34/778 (4.4%) | 4.56 (3.03-6.86) | <0.001 |
| ECS late preterm vs ECS ≥39 | RDS requiring surfactant | 23/261 (8.8%) | 5/778 (0.6%) | 13.71 (5.27-35.70) | <0.001 |
| ECS late preterm vs ECS ≥39 | Any morbidity | 128/261 (49.0%) | 119/778 (15.3%) | 3.21 (2.61-3.94) | <0.001 |
| ECS late preterm vs ECS ≥39 | Hypothermia | 62/261 (23.8%) | 73/778 (9.4%) | 2.53 (1.86-3.45) | <0.001 |
| ECS late preterm vs ECS ≥39 | Hypoglycemia | 6/261 (2.3%) | 4/778 (0.5%) | 4.47 (1.27-15.72) | 0.020 |
| ECS late preterm vs ECS ≥39 | Hyperbilirubinemia requiring treatment | 42/261 (16.1%) | 19/778 (2.4%) | 6.59 (3.90-11.12) | <0.001 |
| ECS late preterm vs ECS ≥39 | Readmission within 28 days | 18/261 (6.9%) | 24/778 (3.1%) | 2.24 (1.23-4.05) | 0.007 |
| ECS early term vs NSVD early term | NICU admission | 304/2308 (13.2%) | 273/4436 (6.2%) | 2.14 (1.83-2.50) | <0.001 |
| ECS early term vs NSVD early term | Any respiratory support | 270/2308 (11.7%) | 108/4436 (2.4%) | 4.81 (3.87-5.97) | <0.001 |
| ECS early term vs NSVD early term | Invasive respiratory support | 14/2308 (0.6%) | 8/4436 (0.2%) | 3.36 (1.41-8.01) | 0.004 |
| ECS early term vs NSVD early term | Transient tachypnea of the newborn | 166/2308 (7.2%) | 132/4436 (3.0%) | 2.42 (1.93-3.02) | <0.001 |
| ECS early term vs NSVD early term | RDS requiring surfactant | 27/2308 (1.2%) | 10/4436 (0.2%) | 5.19 (2.52-10.70) | <0.001 |
| ECS early term vs NSVD early term | Any morbidity | 613/2308 (26.6%) | 616/4436 (13.9%) | 1.91 (1.73-2.11) | <0.001 |
| ECS early term vs NSVD early term | Hypothermia | 379/2308 (16.4%) | 404/4436 (9.1%) | 1.80 (1.58-2.06) | <0.001 |
| ECS early term vs NSVD early term | Hypoglycemia | 39/2308 (1.7%) | 34/4436 (0.8%) | 2.20 (1.40-3.48) | <0.001 |
| ECS early term vs NSVD early term | Hyperbilirubinemia requiring treatment | 90/2308 (3.9%) | 115/4436 (2.6%) | 1.50 (1.15-1.97) | 0.003 |
| ECS early term vs NSVD early term | Readmission within 28 days | 108/2308 (4.7%) | 205/4436 (4.6%) | 1.01 (0.81-1.27) | 0.914 |
| ECS late preterm vs NSVD late preterm | NICU admission | 105/261 (40.2%) | 129/581 (22.2%) | 1.81 (1.47-2.24) | <0.001 |
| ECS late preterm vs NSVD late preterm | Any respiratory support | 94/261 (36.0%) | 81/581 (13.9%) | 2.58 (1.99-3.35) | <0.001 |
| ECS late preterm vs NSVD late preterm | Invasive respiratory support | 11/261 (4.2%) | 4/581 (0.7%) | 6.12 (1.97-19.05) | <0.001 |
| ECS late preterm vs NSVD late preterm | Transient tachypnea of the newborn | 52/261 (19.9%) | 73/581 (12.6%) | 1.59 (1.15-2.19) | 0.005 |
| ECS late preterm vs NSVD late preterm | RDS requiring surfactant | 23/261 (8.8%) | 14/581 (2.4%) | 3.66 (1.91-6.99) | <0.001 |
| ECS late preterm vs NSVD late preterm | Any morbidity | 128/261 (49.0%) | 164/581 (28.2%) | 1.74 (1.45-2.08) | <0.001 |
| ECS late preterm vs NSVD late preterm | Hypothermia | 62/261 (23.8%) | 76/581 (13.1%) | 1.82 (1.34-2.46) | <0.001 |
| ECS late preterm vs NSVD late preterm | Hypoglycemia | 6/261 (2.3%) | 10/581 (1.7%) | 1.34 (0.49-3.64) | 0.590 |
| ECS late preterm vs NSVD late preterm | Hyperbilirubinemia requiring treatment | 42/261 (16.1%) | 64/581 (11.0%) | 1.46 (1.02-2.10) | 0.040 |
| ECS late preterm vs NSVD late preterm | Readmission within 28 days | 18/261 (6.9%) | 55/581 (9.5%) | 0.73 (0.44-1.22) | 0.220 |
| Delivered <39 weeks vs ECS ≥39 | NICU admission | 811/7586 (10.7%) | 69/778 (8.9%) | 1.21 (0.95-1.52) | 0.115 |
| Delivered <39 weeks vs ECS ≥39 | Any respiratory support | 553/7586 (7.3%) | 72/778 (9.3%) | 0.79 (0.62-1.00) | 0.047 |
| Delivered <39 weeks vs ECS ≥39 | Invasive respiratory support | 37/7586 (0.5%) | 8/778 (1.0%) | 0.47 (0.22-1.01) | 0.065 |
| Delivered <39 weeks vs ECS ≥39 | Transient tachypnea of the newborn | 423/7586 (5.6%) | 34/778 (4.4%) | 1.28 (0.91-1.80) | 0.159 |
| Delivered <39 weeks vs ECS ≥39 | RDS requiring surfactant | 74/7586 (1.0%) | 5/778 (0.6%) | 1.52 (0.62-3.74) | 0.361 |
| Delivered <39 weeks vs ECS ≥39 | Any morbidity | 1521/7586 (20.1%) | 119/778 (15.3%) | 1.31 (1.10-1.56) | 0.001 |
| Delivered <39 weeks vs ECS ≥39 | Hypothermia | 921/7586 (12.1%) | 73/778 (9.4%) | 1.29 (1.03-1.62) | 0.024 |
| Delivered <39 weeks vs ECS ≥39 | Hypoglycemia | 89/7586 (1.2%) | 4/778 (0.5%) | 2.28 (0.84-6.20) | 0.095 |
| Delivered <39 weeks vs ECS ≥39 | Hyperbilirubinemia requiring treatment | 311/7586 (4.1%) | 19/778 (2.4%) | 1.68 (1.06-2.65) | 0.024 |
| Delivered <39 weeks vs ECS ≥39 | Readmission within 28 days | 386/7586 (5.1%) | 24/778 (3.1%) | 1.65 (1.10-2.47) | 0.014 |

*Note. Relative risks (RRs) with 95% confidence intervals are shown for prespecified pairwise comparisons. Exposure and reference values are presented as n/N (%).*

**Table S3. Reasons for exclusion by hospital site**

| **Exclusion reason** | **Makassed n (%)** | **Al-Ahli n (%)** | **Total n (%)** |
| --- | --- | --- | --- |
| NSVD at and after 39 weeks | 2654 (51.94%) | 13581 (78.83%) | 16235 (72.68%) |
| Non-elective / urgent delivery | 1437 (28.12%) | 1728 (10.03%) | 3165 (14.17%) |
| Multiple gestation (twins) | 403 (7.89%) | 329 (1.91%) | 732 (3.28%) |
| GA <34 weeks | 384 (7.51%) | 0 (0.00%) | 384 (1.72%) |
| Congenital anomaly / malformation | 53 (1.04%) | 20 (0.12%) | 73 (0.33%) |
| Multiple gestation (triplets) | 48 (0.94%) | 0 (0.00%) | 48 (0.21%) |
| Fetal/neonatal death (IUFD/stillbirth) | 26 (0.51%) | 0 (0.00%) | 26 (0.12%) |
| Assisted vaginal delivery | 0 (0.00%) | 642 (3.73%) | 642 (2.87%) |
| Data error / missing | 23 (0.45%) | 225 (1.31%) | 248 (1.11%) |
| Malpresentation | 0 (0.00%) | 181 (1.05%) | 181 (0.81%) |
| Fetal distress | 0 (0.00%) | 147 (0.85%) | 147 (0.66%) |
| Other / rare reasons | 62 (1.21%) | 149 (0.86%) | 211 (0.94%) |
| Incomplete/undocumented data (reason not recorded) | 43 (0.84%) | 247 (1.43%) | 290 (1.30%) |

*Note. Percentages are presented using the total number of excluded records within each hospital and overall dataset as the denominator, as supplied by the authors.*

**Table S4. Missingness for key exposure, covariate, and outcome variables**

| **Variable** | **Available n/N (%)** | **Missing n/N (%)** | **Missing by hospital** | **Missing by delivery-strategy group** |
| --- | --- | --- | --- | --- |
| Gestational age | 8364/8364 (100.0%) | 0/8364 (0.0%) | 0 missing overall | ECS 34-36: 0/261; NSVD 34-36: 0/581; ECS 37-38: 0/2308; NSVD 37-38: 0/4436; ECS >=39: 0/778 |
| Delivery strategy group | 8364/8364 (100.0%) | 0/8364 (0.0%) | 0 missing overall | ECS 34-36: 0/261; NSVD 34-36: 0/581; ECS 37-38: 0/2308; NSVD 37-38: 0/4436; ECS >=39: 0/778 |
| Neonatal sex | 8324/8364 (99.5%) | 40/8364 (0.5%) | Hospital-specific missingness not available in supplied aggregate output | ECS 34-36: 2/261; NSVD 34-36: 2/581; ECS 37-38: 13/2308; NSVD 37-38: 21/4436; ECS >=39: 2/778 |
| Birth weight | 8318/8364 (99.5%) | 46/8364 (0.5%) | Hospital-specific missingness not available in supplied aggregate output | ECS 34-36: 1/261; NSVD 34-36: 8/581; ECS 37-38: 9/2308; NSVD 37-38: 24/4436; ECS >=39: 4/778 |
| Maternal age | 7959/8364 (95.2%) | 405/8364 (4.8%) | Hospital-specific missingness not available in supplied aggregate output | ECS 34-36: 14/261; NSVD 34-36: 34/581; ECS 37-38: 125/2308; NSVD 37-38: 183/4436; ECS >=39: 49/778 |
| Parity | 8287/8364 (99.1%) | 77/8364 (0.9%) | Hospital-specific missingness not available in supplied aggregate output | ECS 34-36: 5/261; NSVD 34-36: 5/581; ECS 37-38: 35/2308; NSVD 37-38: 27/4436; ECS >=39: 5/778 |
| Gestational diabetes / abnormal OGTT | 8364/8364 (100.0%) | 0/8364 (0.0%) | 0 missing overall | ECS 34-36: 0/261; NSVD 34-36: 0/581; ECS 37-38: 0/2308; NSVD 37-38: 0/4436; ECS >=39: 0/778 |
| Hypertensive disorder | 8364/8364 (100.0%) | 0/8364 (0.0%) | 0 missing overall | ECS 34-36: 0/261; NSVD 34-36: 0/581; ECS 37-38: 0/2308; NSVD 37-38: 0/4436; ECS >=39: 0/778 |
| NICU admission | 8364/8364 (100.0%) | 0/8364 (0.0%) | 0 missing overall | ECS 34-36: 0/261; NSVD 34-36: 0/581; ECS 37-38: 0/2308; NSVD 37-38: 0/4436; ECS >=39: 0/778 |
| Any respiratory support | 8364/8364 (100.0%) | 0/8364 (0.0%) | 0 missing overall | ECS 34-36: 0/261; NSVD 34-36: 0/581; ECS 37-38: 0/2308; NSVD 37-38: 0/4436; ECS >=39: 0/778 |
| TTN | 8364/8364 (100.0%) | 0/8364 (0.0%) | 0 missing overall | ECS 34-36: 0/261; NSVD 34-36: 0/581; ECS 37-38: 0/2308; NSVD 37-38: 0/4436; ECS >=39: 0/778 |
| RDS requiring surfactant | 8364/8364 (100.0%) | 0/8364 (0.0%) | 0 missing overall | ECS 34-36: 0/261; NSVD 34-36: 0/581; ECS 37-38: 0/2308; NSVD 37-38: 0/4436; ECS >=39: 0/778 |
| Hyperbilirubinaemia requiring treatment | 8364/8364 (100.0%) | 0/8364 (0.0%) | 0 missing overall | ECS 34-36: 0/261; NSVD 34-36: 0/581; ECS 37-38: 0/2308; NSVD 37-38: 0/4436; ECS >=39: 0/778 |
| Readmission within 28 days | 8364/8364 (100.0%) | 0/8364 (0.0%) | 0 missing overall | ECS 34-36: 0/261; NSVD 34-36: 0/581; ECS 37-38: 0/2308; NSVD 37-38: 0/4436; ECS >=39: 0/778 |
| Composite neonatal morbidity | 8364/8364 (100.0%) | 0/8364 (0.0%) | 0 missing overall | ECS 34-36: 0/261; NSVD 34-36: 0/581; ECS 37-38: 0/2308; NSVD 37-38: 0/4436; ECS >=39: 0/778 |

Note. Outcome variables were harmonised as binary fields before analysis; missingness in derived outcome indicators reflects the harmonised analytic dataset. Hospital-specific missingness was not available in the supplied aggregate output; where overall missingness was zero, hospital-specific missingness was necessarily zero.

**Table S5. Components of composite neonatal morbidity by prespecified delivery-strategy group**

| **Outcome component** | **ECS 34–36** | **NSVD 34–36** | **ECS 37–38** | **NSVD 37–38** | **ECS ≥39** |
| --- | --- | --- | --- | --- | --- |
| Composite morbidity | 128/261 (49.0%) | 164/581 (28.2%) | 613/2308 (26.6%) | 616/4436 (13.9%) | 119/778 (15.3%) |
| NICU admission | 105/261 (40.2%) | 129/581 (22.2%) | 304/2308 (13.2%) | 273/4436 (6.2%) | 69/778 (8.9%) |
| Any respiratory support | 94/261 (36.0%) | 81/581 (13.9%) | 270/2308 (11.7%) | 108/4436 (2.4%) | 72/778 (9.3%) |
| Invasive respiratory support | 11/261 (4.2%) | 4/581 (0.7%) | 14/2308 (0.6%) | 8/4436 (0.2%) | 8/778 (1.0%) |
| Transient tachypnoea of the newborn | 52/261 (19.9%) | 73/581 (12.6%) | 166/2308 (7.2%) | 132/4436 (3.0%) | 34/778 (4.4%) |
| RDS requiring surfactant | 23/261 (8.8%) | 14/581 (2.4%) | 27/2308 (1.2%) | 10/4436 (0.2%) | 5/778 (0.6%) |
| Hypothermia | 62/261 (23.8%) | 76/581 (13.1%) | 379/2308 (16.4%) | 404/4436 (9.1%) | 73/778 (9.4%) |
| Hypoglycaemia | 6/261 (2.3%) | 10/581 (1.7%) | 39/2308 (1.7%) | 34/4436 (0.8%) | 4/778 (0.5%) |
| Hyperbilirubinaemia requiring treatment | 42/261 (16.1%) | 64/581 (11.0%) | 90/2308 (3.9%) | 115/4436 (2.6%) | 19/778 (2.4%) |
| Readmission within 28 days | 18/261 (6.9%) | 55/581 (9.5%) | 108/2308 (4.7%) | 205/4436 (4.6%) | 24/778 (3.1%) |
| Culture-positive sepsis | 4/261 (1.5%) | 6/581 (1.0%) | 11/2308 (0.5%) | 12/4436 (0.3%) | 5/778 (0.6%) |
| Neonatal death | 1/261 (0.4%) | 1/581 (0.2%) | 1/2308 (0.0%) | 2/4436 (0.0%) | 2/778 (0.3%) |

*Note. Composite neonatal morbidity was defined as at least one recorded component during the birth admission, NICU stay, or 28-day readmission window. Some infants had more than one component, so component totals exceed the composite total.*

**Table S6. Adjusted relative-risk sensitivity analyses**

| **Model / outcome** | **Comparison** | **Adjusted estimate (95% CI)** | **Model N / availability** |
| --- | --- | --- | --- |
| Primary modified Poisson model |  |  |  |
| NICU admission | ECS 34–36 vs ECS ≥39 | aRR 4.52 (3.34-6.12) | 7959 |
| NICU admission | ECS 37–38 vs ECS ≥39 | aRR 1.56 (1.17-2.07) | 7959 |
| Any respiratory support | ECS 34–36 vs ECS ≥39 | aRR 2.81 (2.14-3.69) | 7959 |
| Any respiratory support | ECS 37–38 vs ECS ≥39 | aRR 1.11 (0.87-1.42) | 7959 |
| TTN | ECS 34–36 vs ECS ≥39 | aRR 5.89 (3.68-9.43) | 7959 |
| TTN | ECS 37–38 vs ECS ≥39 | aRR 2.29 (1.50-3.48) | 7959 |
| RDS requiring surfactant | ECS 34–36 vs ECS ≥39 | aRR 13.30 (5.06-34.95) | 7959 |
| RDS requiring surfactant | ECS 37–38 vs ECS ≥39 | aRR 1.88 (0.72-4.89) | 7959 |
| Hyperbilirubinaemia requiring treatment | ECS 34–36 vs ECS ≥39 | aRR 5.74 (3.23-10.20) | 7959 |
| Hyperbilirubinaemia requiring treatment | ECS 37–38 vs ECS ≥39 | aRR 1.73 (1.01-2.98) | 7959 |
| Composite morbidity | ECS 34–36 vs ECS ≥39 | aRR 1.98 (1.70-2.31) | 7959 |
| Composite morbidity | ECS 37–38 vs ECS ≥39 | aRR 1.31 (1.14-1.49) | 7959 |
| Within-gestational-age delivery-mode comparisons |  |  |  |
| NICU admission | ECS 34–36 vs NSVD 34–36 | aRR 1.61 (1.24-2.10) | 794 |
| NICU admission | ECS 37–38 vs NSVD 37–38 | aRR 1.89 (1.58-2.26) | 6436 |
| Any respiratory support | ECS 34–36 vs NSVD 34–36 | aRR 1.87 (1.37-2.54) | 794 |
| Any respiratory support | ECS 37–38 vs NSVD 37–38 | aRR 3.73 (2.95-4.71) | 6436 |
| TTN | ECS 34–36 vs NSVD 34–36 | aRR 1.65 (1.10-2.48) | 794 |
| TTN | ECS 37–38 vs NSVD 37–38 | aRR 2.63 (2.09-3.31) | 6436 |
| RDS requiring surfactant | ECS 34–36 vs NSVD 34–36 | aRR 2.59 (1.25-5.36) | 794 |
| RDS requiring surfactant | ECS 37–38 vs NSVD 37–38 | aRR 4.99 (2.38-10.46) | 6436 |
| Hyperbilirubinaemia requiring treatment | ECS 34–36 vs NSVD 34–36 | aRR 1.08 (0.70-1.65) | 794 |
| Hyperbilirubinaemia requiring treatment | ECS 37–38 vs NSVD 37–38 | aRR 1.05 (0.79-1.41) | 6436 |
| Composite morbidity | ECS 34–36 vs NSVD 34–36 | aRR 1.26 (1.08-1.46) | 794 |
| Composite morbidity | ECS 37–38 vs NSVD 37–38 | aRR 1.22 (1.13-1.33) | 6436 |
| Sensitivity model additionally adjusted for birth weight |  |  |  |
| NICU admission | ECS 34–36 vs ECS ≥39 | aRR 3.25 (2.31-4.57) | 7914 |
| NICU admission | ECS 37–38 vs ECS ≥39 | aRR 1.33 (1.00-1.78) | 7914 |
| Any respiratory support | ECS 34–36 vs ECS ≥39 | aRR 2.04 (1.47-2.81) | 7914 |
| Any respiratory support | ECS 37–38 vs ECS ≥39 | aRR 0.96 (0.74-1.24) | 7914 |
| TTN | ECS 34–36 vs ECS ≥39 | aRR 4.78 (2.93-7.78) | 7914 |
| TTN | ECS 37–38 vs ECS ≥39 | aRR 2.06 (1.35-3.13) | 7914 |
| RDS requiring surfactant | ECS 34–36 vs ECS ≥39 | aRR 6.96 (2.45-19.78) | 7914 |
| RDS requiring surfactant | ECS 37–38 vs ECS ≥39 | aRR 1.42 (0.55-3.66) | 7914 |
| Hyperbilirubinaemia requiring treatment | ECS 34–36 vs ECS ≥39 | aRR 3.96 (2.10-7.47) | 7914 |
| Hyperbilirubinaemia requiring treatment | ECS 37–38 vs ECS ≥39 | aRR 1.46 (0.84-2.55) | 7914 |
| Composite morbidity | ECS 34–36 vs ECS ≥39 | aRR 1.46 (1.23-1.73) | 7914 |
| Composite morbidity | ECS 37–38 vs ECS ≥39 | aRR 1.15 (1.00-1.32) | 7914 |
| Logistic regression sensitivity model |  |  |  |
| NICU admission | ECS 34-36 vs ECS >=39 | aOR 4.68 (3.16-6.93) | Complete-case model |
| NICU admission | ECS 37-38 vs ECS >=39 | aOR 1.26 (0.94-1.68) | Complete-case model |
| Any respiratory support | ECS 34-36 vs ECS >=39 | aOR 3.06 (2.01-4.67) | Complete-case model |
| Any respiratory support | ECS 37-38 vs ECS >=39 | aOR 0.95 (0.70-1.28) | Complete-case model |
| TTN | ECS 34-36 vs ECS >=39 | aOR 4.10 (2.45-6.84) | Complete-case model |
| TTN | ECS 37-38 vs ECS >=39 | aOR 1.45 (0.98-2.14) | Complete-case model |
| RDS requiring surfactant | ECS 34-36 vs ECS >=39 | aOR 6.13 (2.04-18.39) | Complete-case model |
| RDS requiring surfactant | ECS 37-38 vs ECS >=39 | aOR 1.22 (0.46-3.26) | Complete-case model |
| Hyperbilirubinaemia requiring treatment | ECS 34-36 vs ECS >=39 | aOR 3.68 (1.93-7.01) | Complete-case model |
| Hyperbilirubinaemia requiring treatment | ECS 37-38 vs ECS >=39 | aOR 1.19 (0.70-2.01) | Complete-case model |
| Composite morbidity | ECS 34-36 vs ECS >=39 | aOR 2.62 (1.76-3.90) | Complete-case model |
| Composite morbidity | ECS 37-38 vs ECS >=39 | aOR 1.44 (1.12-1.86) | Complete-case model |
| Additional sensitivity analyses requested by reviewers |  |  |  |
| Respiratory composite | Adjusted model | Not estimated | Requires individual-level composite construction |
| Severe composite excluding isolated hypothermia/hyperbilirubinaemia | Adjusted model | Not estimated | Requires individual-level composite construction |
| Antenatal corticosteroid subgroup | Adjusted or stratified model | Not performed | Documentation incomplete/inconsistent across hospitals |

Note. Primary adjusted estimates were obtained using modified Poisson regression with robust variance and adjusted for neonatal sex, maternal age, parity category, gestational diabetes/abnormal OGTT, hypertensive disorder, and hospital site. Birth-weight sensitivity models additionally adjusted for birth weight. Logistic regression models are shown as sensitivity analyses and should not be interpreted as risk ratios. Respiratory-composite, severe-composite, and antenatal-corticosteroid subgroup analyses were not performed because the required uniformly documented individual-level variables were not available in the supplied analytic outputs.

**Supplementary Figures**

**Figure S1. Patient flow diagram**


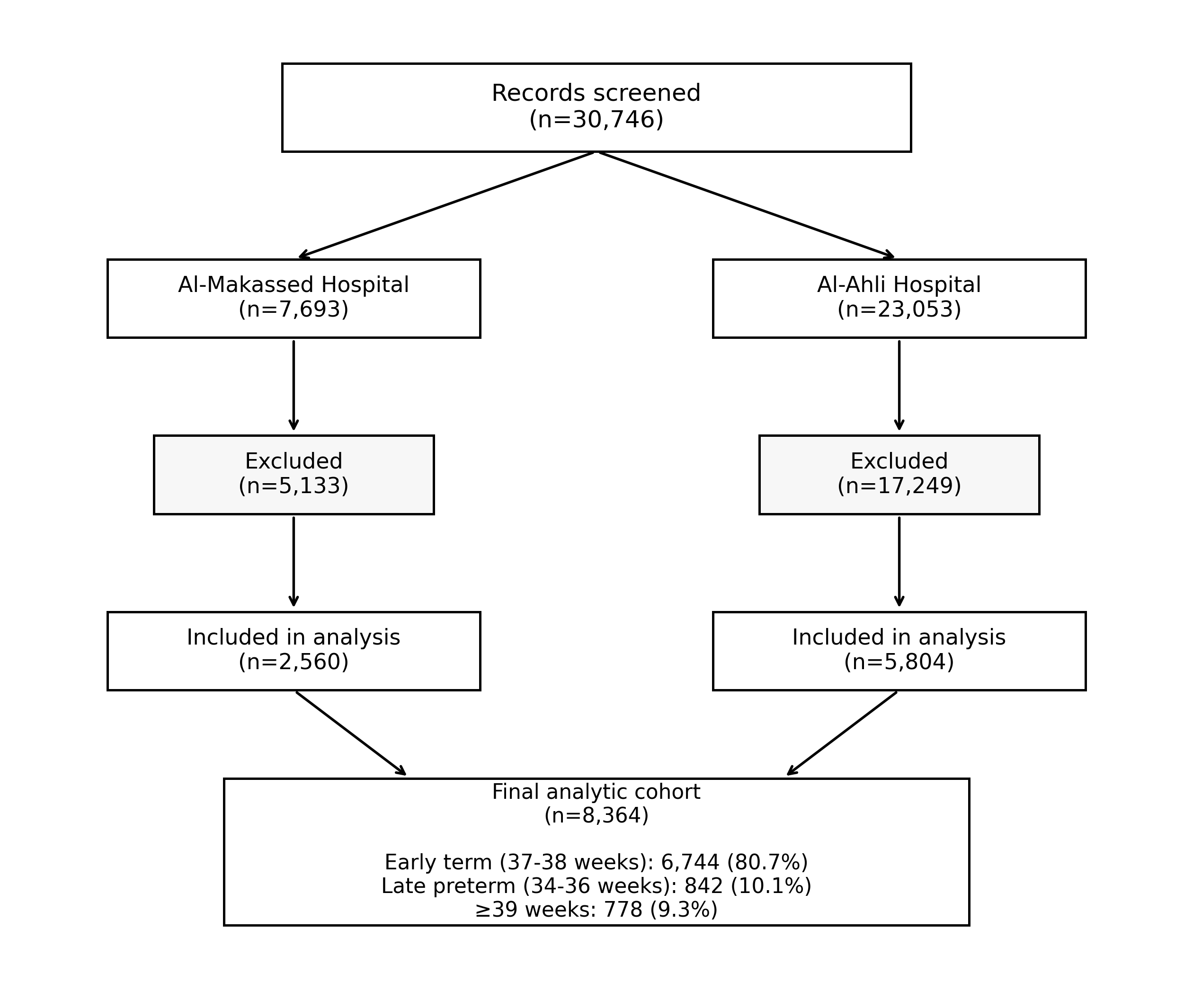


Flow diagram summarising the screening of delivery records from Al-Makassed Hospital and Al-Ahli Hospital (January 2021-December 2024), including exclusions and the final analytic cohort stratified by gestational age group.

**Figure S2. Heatmap of recorded neonatal morbidities across delivery-gestational groups**


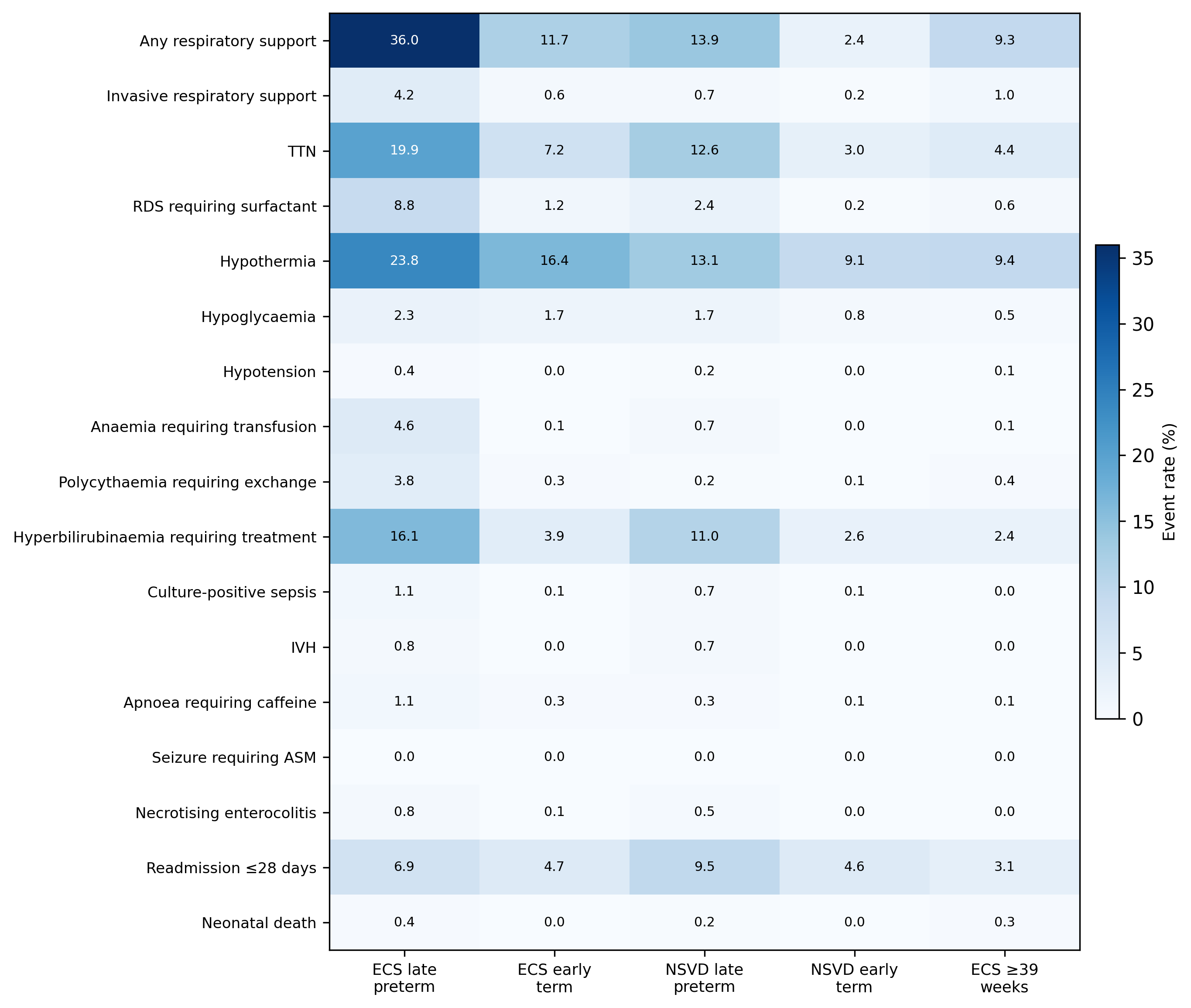


Values within cells are percentages calculated using the total number of neonates in each group as the denominator.

**Figure S3. Birth weight by gestational age and delivery mode**


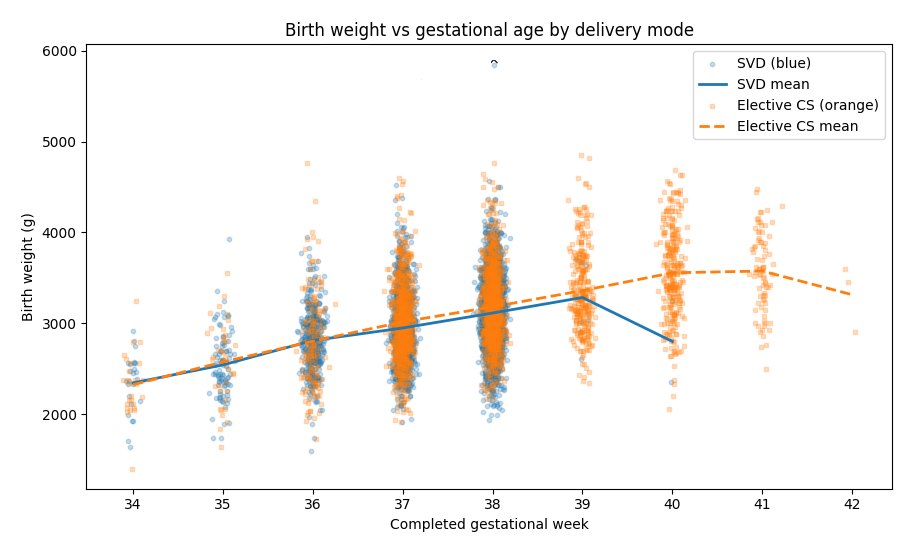


Scatter plot of birth weight across completed gestational weeks stratified by delivery mode. Solid and dashed lines represent week-specific mean birth weight. This figure was preserved from the author-supplied source image.
